# The Influence of an Additional Load on Time and Force Changes in the Ground Reaction Force During the Countermovement Vertical Jump

**DOI:** 10.2478/hukin-2013-0059

**Published:** 2013-10-08

**Authors:** Frantisek Vaverka, Zlatava Jakubsova, Daniel Jandacka, David Zahradnik, Roman Farana, Jaroslav Uchytil, Matej Supej, Janez Vodicar

**Affiliations:** 1 Human Motion Diagnostic Center, University of Ostrava, The Czech Republic.; 2 Department of Physical Education and Sports, VŠB-Technical University of Ostrava, Ostrava, The Czech Republic.; 3 Faculty of Sport, University of Ljulbjana, Ljubljana, Slovenia.

**Keywords:** strength training, SSC, kinetic analysis, force plate, vertical jump

## Abstract

The aim of this study was to determine how an additional load influences the force-vs-time relationship of the countermovement vertical jump (CMVJ). The participants that took part in the experiment were 18 male university students who played sport recreationally, including regular games of volleyball. They were asked to perform a CMVJ without involving the arms under four conditions: without and with additional loads of 10%, 20%, and 30% of their body weight (BW). The vertical component of the ground reaction force (GRF) was measured by a force plate. The GRF was used to calculate the durations of the preparatory, braking, and acceleration phases, the total duration of the jump, force impulses during the braking and acceleration phases, average forces during the braking and acceleration phases, and the maximum force of impact at landing. Results were evaluated using repeated-measures ANOVA. Increasing the additional load prolonged both the braking and acceleration phases of the jump, with statistically significant changes in the duration of the acceleration phase found for an additional load of 20% BW. The magnitude of the force systematically and significantly increased with the additional load. The force impulse during the acceleration phase did not differ significantly between jumps performed with loads of 20% and 30% BW. The results suggest that the optimal additional load for developing explosive strength in vertical jumping ranges from 20% to 30% of BW, with this value varying between individual subjects.

## Introduction

This research focused on the effects of placing an additional load inside a special vest on the countermovement vertical jump (CMVJ). The CMVJ most closely approximates natural jumping, and placing such an additional load does not fundamentally hinder the technique of the jump. The CMVJ is considered to be a suitable tool for both strength training itself and assessing the effectiveness of training. Numerous scientific studies have used jumping to investigate how various factors influence the development of maximal strength and power output during jumping (Bobbert et al., 1996; Bosco and Komi, 1981). Comparisons of the CMVJ and squat jump (SJ) have indicated that training that includes countermovement has a greater effect on the development of explosive strength (Cormie et al., 2009; Walshe et al., 1998). The combination of muscle activities in eccentric contractions at a braking phase during the lowering of the body position and subsequent concentric contractions during body elevation is considered to be the most effective progression method of muscle training, and has been termed the stretch-shortening cycle (SSC) (Bosco and Komi, 1979, 1981; Anderson and Pandy, 1993; Bobbert et al., 1996). Both practical and empirical investigations have shown that applying the SSC increases the effectiveness of strength training.

Quantifying the additional load is a key issue in maximizing muscular strength, which has been investigated in many studies. The amount of an additional load applied in exercises with a barbell is almost always expressed as a percentage of one repetition maximum (1RM, e.g., Cormie et al., 2007; Dugan et al., 2004; McBride et al., 2002; Moss et al., 1997; Stone et al., 2003; Thomas et al., 2007), as the maximum isometric muscle contraction measured at specific positions on tested segments (Kaneko et al., 1983), in units of the load weight in kilograms (Nelson and Martin, 1985; Sheppard et al., 2007), or as a percentage of body weight (BW) (Kraemer and Newton, 1994; Makaruk et al., 2010; Patterson et al., 2009). Quantifying the load as a percentage or 1RM or as the maximum isometric muscle force requires a specific measurement for determining the input value, which is dependent on the difficult-to-control voluntary-contraction force produced by the individual. Moreover, using a barbell as the additional load disturbs the natural character of the jumping movement.

The additional load required to maximize the development of explosive strength has reportedly varied in the range of 0–60% 1RM (Baker et al., 2001; Cormie et al., 2007; Dugan et al., 2004; Stone et al., 2003). For developing maximal muscle strength when jumping, most scientific studies have favored lighter loads in the range of 30–40% 1RM (Cormie et al., 2007; Dugan et al., 2004; Kaneko et al., 1983; McBride et al., 2002). Combining lighter loads with plyometric exercises has been found to be very effective in maximizing explosive strength (Clutch et al., 1983). Loads of approximately 30% BW (Kraemer and Newton, 1994; Patterson et al., 2009) or 30% of the maximum isometric muscle contraction (Kaneko et al., 1983) have been recommended, while very small additional loads such as 5% BW have been found not to produce significant gains relative to a traditional drop jump program (Makaruk et al., 2010). We therefore decided to investigate the effects of additional loads of up to 30% BW.

Increasing the additional load influences the kinematics of the movement (Bosco and Komi, 1981; Sanders et al., 1993) and the magnitudes of the muscle forces produced (Cormie et al., 2007; Dugan et al., 2004). Bobbert and van Ingen Schenau (1988) and Bobbert and van Soest (1994) considered neuromuscular motion control to be the key factor influencing the effectiveness of strength training. Therefore, it is likely that significantly changing the movement performance will affect the efficiency of neuromuscular control of the movement. It can be assumed that increasing the additional load will prolong the duration of the movement and increase the resulting muscle strength. Another important question is whether the magnitude of the additional load will lead to stagnation or a decrease in the produced muscle strength.

The aim of this study was to determine how additional loads affect the vertical component of the ground reaction force during a CMVJ in terms of (a) time changes of particular phases of the jump and (b) magnitudes of the produced forces. The hypothesis tested was that increasing the additional load will prolong particular phases of the CMVJ and increase the force magnitudes.

## Material and Methods

### Participants

Eighteen male university students from the University of Ostrava volunteered to participate in this experiment [age 20.65 ± 1.36 years (mean ± SD), body height (BH) = 1.82 ± 0.06 m, and body weight (BW) 77.33 ± 8.54 kg]. These subjects were playing recreational sports including regular games of volleyball, had experience with performing different jumps, and had not previously participated in strength training. They were informed about the goal of the experiment, and they had a personal interest in its results. Each subject was thoroughly informed about the risks associated with the study and provided written informed consent. The study was approved by the institutional review board of the Pedagogical Faculty of the University of Ostrava.

### Procedures

Each participant performed the experiment in a laboratory during a single day at the time of subjects’ usual sport activity (afternoon). The analyzed movement comprised the CMVJ without upper-limb movement, which were excluded to avoid arm movements influencing the jump data (Hara et al., 2006). The arms were positioned with the elbows along the body and hands placed on the chest. The vertical jump was initiated from the resting standing position, by first lowering the body and then immediately taking off with maximum exertion with the aim to reach maximum jump height (JH). The execution of the CMVJ was standardized during a warm-up and was controlled during the experimental measurement. The warm-up lasted approximately 5 min and consisted of a short run at medium speed (1.5 min), gymnastic exercises targeting at the lower extremities (2 min), and a series of 10 jumps.

The additional load was applied by placing small iron balls inside a special vest worn on the chest, with equal weight distribution on its front and back parts. Additional weights of 10%, 20%, and 30% BW of each individual were used in the experiment.

After entering the laboratory each participant was initially interviewed to obtain a case history using a series of questions about the general health condition, injuries, and levels of physical activity and sports activity. After taking basic body measurements (BW and BH), the weights of the additional loads relative to the BW were calculated. Jump measurements were made 2 min after completing the warm-up exercises. Two jumps were selected for the research of which JH varied in the range of 5% of the best jump.

In most cases only two jumps were needed for each load. The attempt with the maximum JH was included in the statistical analysis for a given load. There was a 1.5-min rest interval between jumps performed with different loads. The effects of practice and fatigue were minimized by using a Latin square to determine the order in which the different loads were applied. A different initial load was applied to each participant (e.g., 0% BW for the first participant, 10% BW for the second participant).

### Data Analysis Procedures

**Figure 1:**
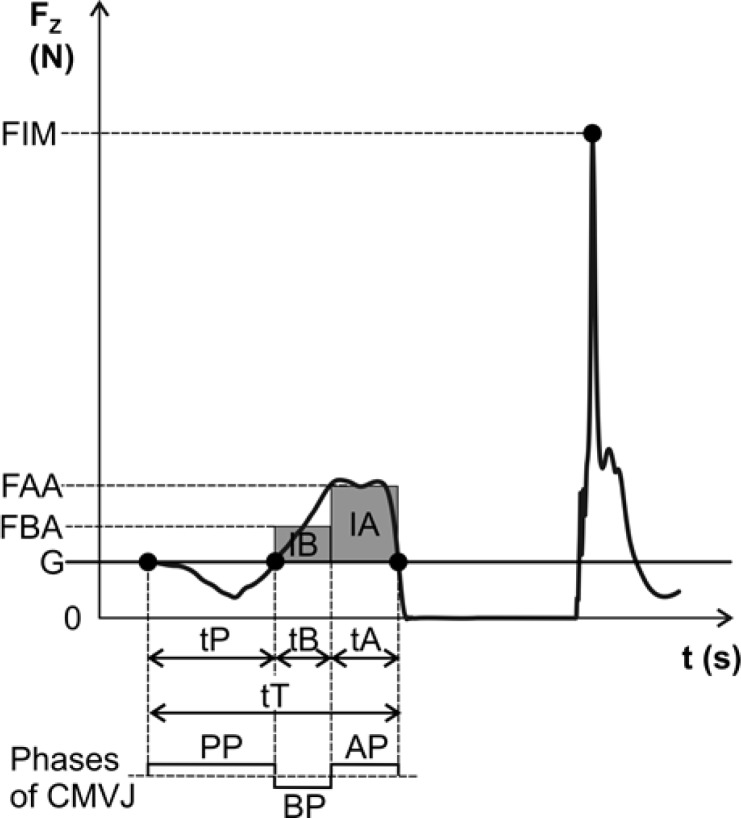
Time course of the ground reaction force during the countermovement vertical jump (CMVJ), and illustration of the individual phases of the jump and measured variables Fz(t) – ground reaction force measured perpendicular to the ground, PP – preparatory phase, BP – braking phase, AP – acceleration phase, tP – duration of the PP, tB – duration of the BP, tA – duration of the AP, tT – total duration of the take-off phase, G – gravitational force acting on the human body (G=m.g, where m is the mass of the subject and g is the gravitational acceleration), IB – force impulse during the BP, IA – force impulse during the AP, FBA – average force during the BP (FBA=IB/tB+G), FAA – average force during the AP (FAA=IA/tA+G), FIM – force of impact at landing

The time course of the reaction force in the vertical direction, *Fz(t)*, produced by the CMVJ was recorded on a force plate (9281CA, sampling frequency 1000 Hz, Kistler Instrumente AG, Winterthur, Switzerland). *Fz(t)* was analyzed by dividing the jump into key phases (Vaverka, 2000): the preparatory phase (PP) corresponds to the initiation of the jump when the body position is lowered, the braking phase (BP) is the deceleration as the body lowers until the body’s center of gravity has a velocity of zero in the downwards direction, and the acceleration phase (AP) is when the body’s center of gravity accelerates in the upwards direction during the vertical take-off (Figure 1). The time course of the take-off was characterized by a set of time variables describing the durations of the particular phases of the take-off and variables associated with the acting forces (Figure 1). The height of jump (JH) was calculated from the magnitude of the acceleration-force impulse (IA) and the weight of the individual according to the formula JH = (IA)^2^ / 2 *m*^2^*g*, where *m* is the BW and *g* is the gravitational acceleration (9.81 m·s^–2^). Selected variables were calculated (Vaverka, 2000) using BioWare (v3.2.6, Kistler Instrumente AG, Winterthur, Switzerland) and MATLAB (Mathworks Inc., Natick, MA).

The interclass correlation coefficient indicated very high reliability of the JH (*r*=0.95–0.99) in all tested variations. The coefficients of reliability for time and the force variables were within the range of *r*=0.68–0.98; they were higher for force variables (most with correlation coefficients *r*>0.90) than for time variables (*r*=0.68–0.94).

### Statistical Analysis

One-way repeated-measures ANOVA (Scheffe’s post-hoc test) was the main statistical method used to evaluate the significance of differences among jumps with different additional loads. It was valid to use this method since the Kolmogorov-Smirnov test of normal data distribution and Cochran’s variance homogeneity test confirmed the basic conditions to use ANOVA. Statistically significant differences as identified by ANOVA with repeated measures are indicated in the tables by asterisks (* *p*<0.05, ** *p*<0.01); the absence of a symbol indicates differences that were not statistically significant. The reliability of the procedure for testing differences between repeated two attempts was calculated using the paired t-test and the interclass Pearson′s correlation coefficient. Data are reported as mean ± SD values. All statistical analyses were performed using Statistica (v8, Statsoft, Inc., Tulsa, OK).

## RESULTS

The JH when jumping with additional loads systematically decreased by about 4 cm when the additional load increased by 10% BW (JH 0.385 m was reached without the load and 0.344 m, 0.309 m, and 0.276 m with the loads 10%, 20%, and 30% BW, respectively).

The JH was computed from the body mass *m* equal to the BW and additional load. All of the differences in the JH were statistically significant.

The changes in the magnitude of the force and time variables exhibited different trends. An increasing additional load increased the force variables, with statistically significant differences in almost all cases (Table 1). The magnitude of the force impulse during the BP (IB) systematically increased, and there were statistically significant differences between the small additional loads (0% and 10% BW) and the maximum load (30% BW). The IA did not differ significantly between additional loads of 20% and 30% BW. We also found that the values of the average forces during the BP (FBA) and AP (FAA) increased significantly with increasing the additional load.

The maximum force at the landing, corresponding to the force at impact (FIM), systematically decreased with increasing additional load, but did not vary significantly.

We found that the values of the time variables changed with different trends at particular phases of the jump (Table 2). With increasing additional load the PP shortened while the BP and AP prolonged, and there were minimal changes in the total duration of the jump. However, the only statistically significant difference in time variables for jumps with different additional loads was in the tA. We found that there was a statistically significant prolongation of the AP between jumps without an additional load and jumps with additional loads of 20% and 30% BW. The total take-off duration changed irregularly and statistically insignificantly when increasing the additional load. The increasing time of the acceleration phase and decreasing time of the preparatory phase contributed to no significant changes in tT.

## Discussion

Additional loads from 10% to 30% BW were chosen based on the conclusions drawn from previous studies (Kraemer and Newton, 1994; Patterson et al., 2009; Wilson et al., 1993). We were interested in how the duration and magnitude of forces associated with the CMVJ change for additional loads within a specific range. Consistent with previous findings (Nelson and Martin, 1985), we found that the JH decreased systematically with an increasing load. This finding was expected given the simple relationship between the JH, BW, and IA. Increasing the weight of the subject by adding an additional load of 10–30% BW cannot be compensated for a relatively small increase in IA resulting in a lower JH. Therefore, the JH calculated from BW and the load cannot be used as a criterion for the effectiveness of strength training on jumping.

We could observe the change in the JH by the effect of an additional load relative to the obtained values of IA and BW without an additional load (Sheppard et al., 2008). The JH was computed based on measured IA and m = BW without an additional load. The results clearly showed that the JH increased significantly with increasing the magnitude of the additional load (from JH = 0.385 without an additional load to 0.416m, 0.445m, and 0.466m with the load of 30% BW). However, an additional load of 30% BW increased the JH by only 2 cm and there was no significant difference between the JHs for additional loads of 20% and 30% BW.

This study found that increasing the additional load systematically increased strength variables during the BP and AP of the CMVJ (i.e., FBA, IB, FAA, and IA; Table 1). We were especially interested in the variables explaining these trends. The key variable influencing the jump behavior is IA: its size systematically and statistically significantly increased from jumping with no load up to jumping with an additional load of 30% BW, with no significant difference for loads of 20% and 30% BW. The value of IB differed significantly only between an additional load of 30% BW and smaller loads. Increasing the additional load significantly affected increasing the magnitudes of FBA and FAA. For the time variables, we found statistically significant changes only for tA between the lowest (no load and 10% BW) and highest (20% and 30% BW) loads. These results suggest that the AP of the jump is significantly prolonged for a load of 20% BW.

We then attempted to quantify how increasing an additional load increases the magnitudes of the force variables and changes the durations of particular phases of the jump. Thus, we normalized the data so that they were presented as percentages relative to jumping without an additional load in order to facilitate comparison of trends amongst individual variables (Table 3). Increasing an additional load increased all of the measured strength variables, with the greatest for a load of 30% BW: IA increased by 10%, IB by 17%, FBA by 22%, and FAA by 12%. It was found that the FIM changed only slightly, with the largest load actually leading to a decrease of up to 5%. Moreover, the heaviest additional load prolonged the BP by 19% and increased tA by 15%.

The magnitude of the additional load that causes a statistically significant prolongation of the movement or when the measured force stops increasing or starts decreasing could be used to determine the optimal additional load for developing explosive strength in jumps. Increasing the additional load by up to 30% BW increased FBA and FAA, which means that such an additional load is suitable for increasing the force, as stated in the literature (Dugan et al., 2004; Kraemer and Newton, 1994). The most important variable for influencing the final characteristics of the jump, IA, did not vary significantly between additional loads of 20% and 30% BW. Also, tA was significantly longer for an additional load of 20% BW than for one of 10% BW. This means that the duration of a jump changes significantly for loads heavier than 10% BW. Moreover, the lack of a statistically significant difference between IA values for jumps with additional loads of 20% and 30% BW suggests the presence of a stagnation mechanism.

**Figure 2:**
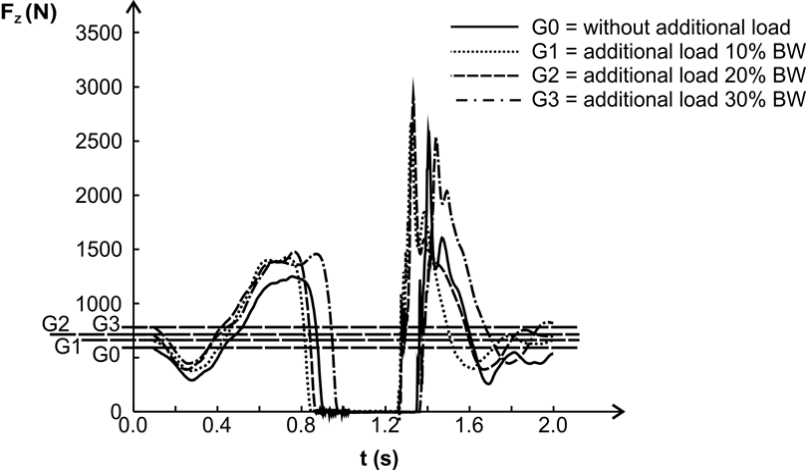
Time course of the Fz(t) curves during the CMVJ for different magnitudes of additional loads. Data of an individual

The graphical representation of the measured Fz (t) in jumps with different additional loads for one of the research participants clearly demonstrates the relations between the magnitude of the force produced and the durations of the various CMVJ phases (Figure 2). The maximum force during the AP increases with an additional load until it reaches 20% BW, while for a load of 30% BW but the AP is significantly prolonged. Therefore, in terms of fulfilling the requirement for consistency of the duration of the movement and a stagnation or decrease of the produced force magnitude, an additional load of 20% BW appears optimum for this individual.

Comparison of the magnitudes of changes in the IA between jumps with additional loads of 20% and 30% BW in individual subjects produced interesting results. We found that relative to an additional load of 20% BW, one of 30% BW decreased the IA in four subjects, produced minimal differences in six subjects, and increased the IA in eight subjects. In contrast, a significant prolongation of the tA was found in most of the study participants. The presented example also demonstrates that the optimum load for an individual depends on the subject’s training (Dugan et al., 2004). In real training, it would be best to decide the load magnitude according to the actual predisposition and training level of an individual. The research results demonstrate that the employed method of analyzing the time course and force of the measured Fz(t) function could be used to individualize the magnitude of the additional load that would maximize the effectiveness of training. It appears that as a general rule an additional load of 20–30% BW is the most suitable for optimizing muscle performance when jumping.

## Conclusions

Gradually increasing an additional load within the range of 10–30% BW systematically prolonged braking and accelerating phases of the counter movement vertical jump, but because the preparation phase shortened, the total duration of the jump did not change. The magnitudes of all force variables increased as the additional load increased up to 30% BW. The time of the acceleration phase significantly increases from the additional load of 20% BW compared with smaller loads of 0% and 10% BW. The magnitude of the force impulse in acceleration and braking phase between loads of 20% and 30% BW stagnated. The results indicate that there are statistically significant changes in the key phases of the jump when applying additional loads of 20% BW (which extends the duration of the acceleration phase of the jump) and 30% BW (which results in stagnation of the force impulse in the acceleration phase). The collected data suggest that the optimum additional load for enhancing muscle strength in jumping ranges from 20% to 30% BW, and that such loads lead to significant changes in the duration of the key phase of the jump and the magnitude of the force impulse in the acceleration phase. Moreover, the strength and training level of an individual need to be considered when determining the optimal additional load to be used in jump-based strength training.

## Figures and Tables

**Table 1:** Differences in countermovement vertical jumps (CMVJs) for different additional loads. Force variables, men, n = 18

Additional load (%BW)	Variable	Mean	SD	Significance of differences		
				Additional load (%BW)		
				0	10	20
0	IB (Ns)	80.62	23.46			
10		79.53	24.23	–		
20		89.54	28.67	–	–	
30		94.03	30.25	^**^	^**^	–

0	IA (Ns)	212.3	27.33			
10		220.2	26.87	^**^		
20		227.9	29.32	^**^	^**^	
30		233.1	27.98	^**^	^**^	–

0	FBA (N)	1150.2	190.0			
10		1177.1	182.2	–		
20		1305.9	228.0	^**^	^**^	
30		1405.1	241.3	^**^	^**^	^*^

0	FAA (N)	1543.9	278.6			
10		1595.1	237.8	–		
20		1661.8	242.9	^**^	^*^	
30		1733.4	250.1	^**^	^**^	^**^

0	FIM (N)	5192.0	1448.1			
10		5189.5	1444.7	–		
20		5071.3	2006.2	–	–	
30		4935.1	1190.6	–	–	–

IB – force impulse during the braking phase, IA – force impulse during the acceleration phase, FBA – average force during the braking phase, FAA – average force during the acceleration phase, FIM – force of impact at landing

* p < 0.05,

** p < 0.01

**Table 2:** Differences in countermovement vertical jumps (CMVJs) for different additional loads. Time variables, men, n = 18

Additional load (%BW)	Variable	Mean	SD	Significance of differences		
				Additional load (%BW)		
				0	10	20
0	tP (s)	0.478	0.124			
10		0.497	0.135	–		
20		0.461	0.116	–	–	
30		0.468	0.112	–	–	–

0	tB (s)	0.216	0.053			
10		0.243	0.063	–		
20		0.246	0.068	–	–	
30		0.256	0.096	–	–	–

0	tA (s)	0.286	0.072			
10		0.303	0.071	–		
20		0.315	0.068	^**^	–	
30		0.328	0.083	^**^	^*^	–

0	tT (s)	0.980	0.191			
10		1.043	0.175	–		
20		1.022	0.182	–	–	
30		1.052	0.213	–	–	–

tP – duration of the preparatory phase, tB – duration of the braking phase, tA – duration of the acceleration phase, tT – total duration of the jump

* p < 0.05,

** p < 0.01.

**Table 3:** Values of the measured variables in jumps performed with different additional loads, expressed as percentages relative to jumping without an additional load (normalized to 100%). Men, n = 18

Additional load (%BW)	Jump height (%)	Force variables (%)					Time variables (%)			

		IB	IA	FBA	FAA	FIM	tP	tB	tA	tT
0	100	100	100	100	100	100	100	100	100	100
10	89	99	104	102	103	100	104	113	106	106
20	80	111	107	114	108	98	96	114	110	104
30	72	117	110	122	112	95	98	119	115	107

JH – jump height, IB – force impulse during the braking phase, IA – force impulse during the acceleration phase, FBA – average force during the braking phase, FAA – average force during the acceleration phase, FIM – force of impact at landing, tP – duration of the preparatory phase, tB – duration of the braking phase, tA – duration of the acceleration phase, tT – total duration of the jump
